# Editorial: Identity work in coaching: new developments and perspectives for business and leadership coaches and practitioners

**DOI:** 10.3389/fpsyg.2024.1517877

**Published:** 2024-12-16

**Authors:** Dorota Bourne, Kurt April, Babar Dharani

**Affiliations:** ^1^Henley Business School, University of Reading, Reading, United Kingdom; ^2^Graduate School of Business, University of Cape Town, Cape Town, South Africa

**Keywords:** coaching, identity, identity work, leadership, leadership development

As the world becomes more complex and fast-paced, leaders, in particular, face unique challenges in aligning their personal values with the roles they are required to play in organizations. This alignment is often where confidential, individually-tailored coaching proves to be most valuable (Szekely et al., 2024). We differentiate identity development (Kragt and Day, 2020) from identity work (Snape, 2021), the latter referring to the self-processing competence and requisite sensemaking through which individuals actively construct, revise, or reinforce their identities in response to life changes or professional transitions. Coaching helps individuals navigate the related identity questions, identity crises, identity negotiation, and identity transitions that emerge from life changes and professional transitions by creating safe, reflective spaces where they can dynamically explore who they are (self-awareness and epistemic cognition) and how they wish to present themselves to the world (instrumental repertoire). This process/work is not only about resolving inner conflict but also about fostering personal growth and enhancing leadership capabilities that are grounded in self-insight, self-acceptance, self-confidence and ultimately authentic behaviors.

Recent research on coaching and identity also highlights the complex interplay between coaches' and leaders' identities and their professional practices. Studies have explored how coaches approach identity construction through life narratives (Butcher, 2012) and how coaching can challenge and reshape professional identities (Byrnes et al., 2019)—including those of the coaches themselves and the meanings they ascribe to their roles (Pope et al., 2014), as well as the transition by experienced professionals from a variety of backgrounds into coaching roles (Evans and Lines, 2014). Importantly, the coaches' positionality in the coaching space, their maturity in regulating their self-interests, and their epistemological orientations, are all critical elements of the coaching relationship. The concept of a ‘joint coaching identity' has been investigated, emphasizing the importance of power dynamics and social identity theory in coaching relationships (Lai and Smith, 2021).

This Research Topic includes five articles that shed light on various dimensions of identity work in coaching.

The first article, “*Exploring the Role of Dynamic Presencing in Fostering Transformative Leadership Development During Disruptive Times*” (Proches et al.), introduces Dynamic Presencing as a method for cultivating inner leadership capabilities through self-awareness. This paper focuses on how identity work within group coaching can help leaders navigate VUCA conditions of today's world. By integrating personal transformation with leadership identity development, the article offers novel insights into how coaching can empower leaders to manage disruption more effectively and how identity work through Dynamic Presencing deepens leadership transformation, confidence and resourcefulness. The authors offer a novel coaching method that links identity work with leadership adaptability.

In “*The MAP (Me-as-a-Process) Coaching Model: A Framework for Coaching Women's Identity Work in Voluntary Career Transitions*” (Snape), the focus is on women undergoing voluntary career transitions. The article introduces the MAP Coaching Model, which addresses the emotional and psychological complexities involved in these transitions and helps coaches guide clients through the identity shifts that accompany career changes. The model identifies four stages of identity work and offers coaches a structured framework to support their clients. This paper contributes to the growing body of literature on gender, career transition, and coaching, emphasizing the importance of identity-focused coaching for women in leadership roles.

Next, “*Social Difference and Relational Coaching: Finding New Freedoms in Working with Identity*” (Tawadros et al.) explores how intersectional social identity differences, such as race, gender, and class, affect the coaching relationship and identity. The authors argue that conventional coaching models often narrowly perpetuate Western ideals of leadership, and introduces the concept of the “implicated subject,” a framework for understanding how social power dynamics influence identity. The study draws on real-life coaching examples to illustrate how addressing social differences directly in coaching can enrich the working alliance between coach and coachee. The authors conclude that a relational approach to coaching, which incorporates discussions of social difference, can lead to deeper insights and more meaningful identity work.

In “*Decolonial Identities in the Leadership Coaching Space: Against Neoliberal Leader Identity Regulation*” (Seyama-Mokhaneli and Belang), the focus shifts to how coaching can resist neoliberal identity frameworks that often dominate leadership development. The authors argue that conventional coaching models often perpetuate Western ideals of leadership that do not resonate with individuals from marginalized backgrounds. Drawing on Black feminist pedagogy, this paper presents a decolonial, critically conscious approach to coaching, emphasizing how honoring decolonial identities can foster authentic leadership development. Using qualitative research, the study demonstrates how a decolonial coaching framework can help leaders from Black and Indigenous communities develop authentic leadership identities that are rooted in their cultural heritage.

The final paper, “*Exploring Identity in Coaching: Insights into Coaches' Understanding and Approach*” (Lazarus), examines how executive coaches engage with identity issues during their sessions. The study, based on interviews with 14 executive coaches, uncovers a significant gap in coach education regarding identity work and calls for a more systematic/structured approach and advocates for coach education programs to include identity work as a core competency. The findings underline the necessity for a systematic approach to help coachees navigate identity conflicts, especially in today's rapidly changing world.

The articles featured in this Research Topic collectively highlight the central role of identity in coaching, particularly in contexts of leadership and personal mastery. As individuals navigate and make sense of complex social, professional, and cultural environments, their identities are constantly being reshaped. Coaching provides a crucial space for this identity work, offering individuals the opportunity to explore who they are, who they want to become, and how they can align their identities with their professional roles.

Research consistently shows that individuals who engage in identity work through coaching are more likely to succeed in their personal and professional lives. This success is not just about achieving goals but also about cultivating a deeper understanding of themselves and their place in the world. As coaches continue to develop new tools and frameworks for identity work, it is clear that this area will remain a critical aspect of coaching practice.
